# Supplementary material for: The Impact of Diagnostic Ureteroscopy Prior to Radical Nephroureterectomy on Oncological Outcomes in Patients with Upper Tract Urothelial Carcinoma: A Comprehensive Systematic Review and Meta-Analysis
Source: J Clin Med. 2021 Sep 16;10(18):4197. doi: 10.3390/jcm10184197 (PMC8465597; doi:10.3390/jcm10184197)
Supplement: Supplementary file 1 [file jcm-10-04197-s001.zip › jcm-1322133-supplementary.pdf]

Table S1. Technical details of ureteroscopy and radical nephroureterectomy in included studies.

Author, Year [reference]	URS Technique	Time between URS and RNU	Surgical Approach	Bladder Cuff Excision
Baboudjian et al. 2020 [11]	Flexible URS<br>Use of Peel-Away ureteral sheath Ch 9–10, drainage with mono-J and bladder catheter	Mean 47 days	Open/laparoscopic RNU	URS (+): 70/70 (100%)<br>URS (-) : 23/23 (100%)
Boorjian et al. 2005 [12]	Rigid URS	Mean 28 days	Open/laparoscopic RNU	NR
Chung et al. 2020 [13]	Flexible/rigid URS	NR	Open/laparoscopic/robotic RNU	URS (+): 226/226 (100%)<br>URS (-) : 227/227 (100%)
Hendin et al. 1999 [14]	Rigid URS	NR	NR	URS (+): 48/48 (100%)<br>URS (-): 48/48 (100%)
Ishikawa et al. 2010 [15]	Flexible/rigid URS	NR	Open/laparoscopic RNU	URS (+): 55/55 (100%)<br>URS (-): 153/153 (100%)
Izol et al. 2021 [16]	Flexible/rigid URS	Median 30 days	Open/laparoscopic/robotic RNU	URS (+): 95/95 (100%)<br>URS (-): 99/99 (100%)
Lee HY et al. 2018 [17]	NR	NR	Open/laparoscopic RNU, segmental resection	URS (+): 206/206 (100%)<br>URS (-): 296/296 (100%)
Lee JK et al. 2016 [18]	NR	Group 1: Same day<br>Group 2: Median 5 days	Open/laparoscopic/robotic RNU	NR
Liu et al. 2016 [19]	NR	NR	Open/laparoscopic RNU	URS (+): 81//81 (100%)<br>URS (-): 583/583 (100%)
Luo et al. 2013 [20]	Rigid URS	NR	NR	URS (+): 115/115 (100%)<br>URS (-): 281/281 (100%)
Ma et al. 2019 [21]	Flexible/rigid URS	NR	Open/laparoscopic	NR
Nison et al. 2013 [22]	Rigid URS	Median 79.5 days	Open/laparoscopic RNU	NR
Sankin et al. 2016 [23]	NR	Median 35 days	NR	NR
Sharma et al. 2021 [24]	NR	NR	NR	URS (+): 452/567 (79.7%)*<br>URS (-) : 101/210 (48.1%)
Sung et al. 2015 [25]	Flexible/rigid URS	Median 16 days	Open/laparoscopic RNU	URS (+): 282 (100%)<br>URS (-): 348 (100%)
Yoo et al. 2017 [26]	Semi-rigid/flexible URS	NR	Open/laparoscopic/robotic RNU	URS (+): 69/69 (100%)<br>URS (-): 318/318 (100%)

* Statistically significant difference between URS (+) and URS (-) groups; Abbreviations: Ch = Charriere; NR = not reported; RNU = radical nephroureterectomy; URS = ureteroscopy.

Table S2 Characteristics of intravesical recurrences in included studies.

Author, year [reference]	Group	Number of intravesical recurrences, n (%)	Pathological stage of bladder recurrence	Pathological grade of bladder recurrence
**Baboudjian** *et al.* 2020 [11]	**URS (+)** n = 70	41 (59.0)*	<T2: 41 (100.0)<br>≥T2: 0 (0.0)	LG: 23 (56.1)<br>HG: 18 (43.9)
	**URS (-)** n = 23	6 (26.0)	<T2: 6 (100.0)<br>≥T2: 0 (0.0)	LG: 4 (66.7)<br>HG: 2 (33.3)
**Chung** *et al.* 2020 [13]	**URS (+)** n = 226	99 (43.8)*	NR	NR
	**URS (-)** n = 227	61 (26.9)	NR	NR
**Ishikawa** *et al.* 2010 [15]	**URS (+)** n = 55	NA	NR	NR
	**URS (-)** n = 153	NA	NR	NR
**Izol** *et al.* 2021 [16]	**URS (+)** n = 95	37 (38.9)*	NR	NR
	**URS (-)** n = 99	17 (17.2)	NR	NR
**Lee HY** *et al.* 2018 [17]	**URS (+)** n = 206	NA	NR	NR
	**URS (-)** n = 296	NA	NR	NR
**Lee JK** *et al.* 2016 [18]	**URS (+)** n = 74[#]	29 (39.2)*	NR	NR
	**URS (-)** n = 30	5 (16.7)	NR	NR
**Liu** *et al.* 2016 [19]	**URS (+)** n = 81	NA	NR	NR
	**URS (-)** n = 583	NA	NR	NR
**Luo** *et al.* 2013 [20]	**URS (+)** n = 115	47 (40.9)*	NR	NR
	**URS (-)** n = 281	78 (27.8)	NR	NR
**Ma** *et al.* 2019 [21]	**URS (+)** n = 110	NA	NR	NR
	**URS (-)** n = 53	NA	NR	NR

**Sankin** *et al.* 2016 [23]	**URS (+)** n = 144	NA	NR	NR
	**URS (-)** n = 57	NA	NR	NR
**Sharma** *et al.* 2021 [24]	**URS (+)** n = 567[##]	157 (27.7)	<cT2: 147 (93.6)*<br>≥cT2: 10 (6.4)	NR
	**URS (-)** n = 210	47 (22.4)	<cT2: 37 (78.7)<br>≥cT2: 10 (21.3)	NR
**Sung** *et al.* 2015 [25]	**URS (+)** n = 282	NA	NR	NR
	**URS (-)** n = 348	NA	NR	NR
**Yoo** *et al.* 2017 [26]	**URS (+)** n = 69	NA	NR	NR
	**URS (-)** n = 318	NA	NR	NR

[#] data is presented for patients who underwent URS regardless of following RNU time; [##] data is presented for patients who underwent URS regardless of biopsy status ; * statistically significant difference between URS (+) and URS (-) groups; Abbreviations: NA = not applicable; NR = not reported; URS = ureteroscopy.

Table S3. Results of sensitivity analyses.

Author, year [reference]	HR [95% CI] after study exclusion	P - value	Heterogeneity I^2 (%)
**Intravesical recurrence-free survival**			
All studies	**1.44 [1.29, 1.61]**	**<0.001**	**35%**
Baboudjian *et al.* 2020 [11]	1.43 [1.28 -1.60]	<0.001	27%
Chung *et al.* 2020 [13]	1.45 [1.29, 1.63]	<0.001	39%
Ishikawa *et al.* 2010 [15]	1.48 [1.32, 1.66]	<0.001	30%
Izol *et al.* 2021[16]	1.41 [1.27, 1.58]	<0.001	16%
Lee HY *et al.* 2018 [17]	1.48 [1.32, 1.67]	<0.001	32%
Lee JK *et al.* 2016 [18]	1.44 [1.29, 1.60]	<0.001	38%
Liu *et al.* 2016 [19]	1.43 [1.27, 1.60]	<0.001	38%
Luo *et al.* 2013 [20]	1.45 [1.29, 1.62]	<0.001	39%
Ma *et al.* 2019 [21]	1.45 [1.30, 1.62]	<0.001	39%
Sankin *et al.* 2016 [23]	1.42 [1.27, 1.59]	<0.001	29%
Sharma *et al.* 2021 [24]	1.47 [1.31, 1.66]	<0.001	41%
Sung *et al.* 2015 [25]	1.42 [1.26, 1.60]	<0.001	38%
Yoo *et al.* 2017 [26]	1.46 [1.30, 1.64]	<0.001	38%
**Cancer-specific survival**			
All studies	**0.94 [0.75, 1.19]**	**0.63**	**29%**

Chung *et al*. 2020 [13]	1.00 [0.78, 1.29]	0.98	23%
Ishikawa *et al*. 2010 [15]	1.02 [0.79, 1.31]	0.89	0%
Lee HY *et al*. 2018 [17]	0.84 [0.63, 1.11]	0.21	16%
Luo *et al*. 2013 [20]	0.90 [0.70, 1.16]	0.43	34%
Ma *et al*. 2019 [21]	0.91 [0.71, 1.17]	0.46	33%
Nison *et al*. 2013 [22]	0.96 [0.74, 1.23]	0.72	40%s
Sankin *et al*. 2016 [23]	0.97 [0.75, 1.25]	0.82	38%
**Overall survival**			
All studies	**0.94 [0.75, 1.17]**	**0.56**	**47%**
Boorjijan *et al*. 2005 [12]	0.90 [0.72, 1.12]	0.35	40%
Chung *et al*. 2020 [13]	1.06 [0.84, 1.34]	0.65	0%
Hendin *et al*. 1999 [14]	0.93 [0.74, 1.16]	0.50	55%
Lee HY *et al*. 2018 [17]	0.87 [0.67, 1.14]	0.32	53%
Ma *et al*. 2019 [21]	0.91 [0.72, 1.14]	0.41	52%
Sankin *et al*. 2016 [23]	0.98 [0.77, 1.25]	0.90	52%
Sharma *et al*. 2021 [24]	0.92 [0.73, 1.16]	0.48	55%
**Metastases-free survival**			
All studies	0.91 [0.74, 1.12]	0.37	0%
Hendin *et al*. 1999 [14]	0.87 [0.70, 1.08]	0.21	0%
Lee HY *et al*. 2018 [17]	0.85 [0.67, 1.09]	0.21	4%
Luo *et al*. 2013 [20]	0.99 [0.78, 1.25]	0.91	0%
Nison *et al*. 2013 [22]	0.94 [0.75, 1.19]	0.62	17%
Sankin *et al*. 2016 [23]	0.91 [0.74, 1.13]	0.41	24%

Abbreviations: CI = confidence interval; HR = hazard ratio.
